# Internet-based educational intervention to prevent risky sexual behaviors in Mexican adolescents: study protocol

**DOI:** 10.1186/s12889-016-2990-4

**Published:** 2016-04-18

**Authors:** Svetlana V. Doubova, Claudia Infante-Castañeda, Ricardo Pérez-Cuevas

**Affiliations:** Epidemiology and Health Services Research Unit, CMN Siglo XXI, Mexican Institute of Social Security, Av. Cuauhtemoc 330, Mexico City, 06720 Mexico; Instituto de Investigaciones Sociales, Universidad Nacional Autónoma de México, México City, Mexico; Hospital Infantil de Mexico Federico Gomez, Calle Doctor Marquez 162 Colonia Doctores, Mexico City, Pc: 06720 Mexico

**Keywords:** Adolescents, Internet-based educational intervention, Prevention, Risky sexual behaviors

## Abstract

**Background:**

Risky sexual behaviors of adolescents in Mexico are a public health problem; 33.4 % of adolescent girls and 14.7 % of boys report not having used any protection at their first intercourse. The fertility rate is 77 births/1000 girls aged 15–19 years. The infrequent contact of adolescents with health services and the limited extent of school sex and reproductive health education require the support of innovative strategies. The objective of this paper is to present the design of an internet-based educational strategy to prevent risky sexual behaviors in Mexican adolescents.

**Methods:**

A field trial with intervention and comparison group and with ex-ante and ex-post measurements will be conducted in two public secondary schools. Adolescents between 14 and 15 years of age will participate. The intervention will be conducted in one school and the second school will serve as a comparison group where the investigators will observe the usual sex education provided by the school. The intervention will be delivered using an internet web page that includes four educational sessions provided during a 4 week period. Follow-up will last 3 months. Information on the study variables will be obtained through an Internet-based self-applied questionnaire and collected on three occasions: 1) when the adolescents enter the study (baseline), 2) once the intervention is completed (at 1 month) and 3) after 3 months of follow-up (at the fourth month). There will be three outcome variables: 1) knowledge in regard to sexually transmitted infections, 2) attitudes regarding condom use, and 3) self-efficacy toward consistent condom use. The generalized linear model will be used to assess changes in each outcome variable controlling for baseline measures and for study covariates.

**Discussion:**

The design and evaluation of an Internet-based educational strategy to prevent risky sexual behaviors in Mexican adolescents is important in order to provide a new, large-scale, easily implemented preventive tool.

**Trial registration:**

The study was registered at the ClinicalTrials.gov ID: NCT02686736.

## Background

Adolescence is a stage of life between 10 and 19 years of age in which young people experience complex biological, psychological and social changes. Some of these changes encompass rapid physical growth, sexual maturation, manifestation of sexual interest and initiation of sexual life [1] that usually begins between 15 and 19 years of age [2]. From a demographic perspective, adolescents represent a significant proportion of the population. In Latin America there are 148 million adolescents, accounting for 30 % of the total population [3].

Adolescents often engage in risky sexual behaviors that include unprotected sex, lack of contraceptive use, and multiple sexual partners. A recent multi-country survey representative of attitudes of young persons toward sex and contraception that included 5426 adolescents from 26 countries reported that, between 2009 and 2011, the number of young people having unprotected sex with a new partner increased substantially. For instance, in France, this increase was 111 % [4]. Furthermore, the proportion of adolescents with multiple sexual partners in the last 12 months increased to 41 % in developed countries and 58 % in developing countries [5].

These risky behaviors are associated with a high rate of unplanned pregnancies and sexually transmitted infections. According to the World Health Organization, 16 million girls aged between 15 and 19 years give birth every year with 95 % of these births occurring in “developing countries” [6]. Additionally, adolescents represent almost 50 % of new cases of sexually transmitted infections (STIs) [7]. Other important health consequences are unsafe abortions, pregnancy complications, infertility, significant psychosocial consequences and premature deaths [8].

In Mexico, risky sexual behaviors of adolescents are a public health problem. The 2012 Health and Nutrition National Survey (ENSANUT 2012 Spanish acronym) reported that 33.4 % of adolescent girls and 14.7 % of boys did not use any protection at the first intercourse [9]. Of sexually active female adolescents, 51.9 % had been pregnant; 5.7 % of adolescents from disadvantaged urban areas had herpes simplex virus, and 18 % had human papilloma virus [8]. The National Center for the Prevention and Control of Human Immunodeficiency Virus Infection (HIV) and Acquired Immune Deficiency Syndrome (AIDS) in Mexico has stressed that HIV is an important problem; ~180,000 Mexicans live with HIV. In 90 % of the cases, it was transmitted through sexual contact and in 94.1 % was due to inconsistent and incorrect condom use. In 2012, there were 9300 new cases registered of HIV infection; 5 % were adolescents [10]. Furthermore, ENSANUT 2012 reported that slightly more than half of Mexican adolescents had some knowledge about HIV transmission, symptoms and methods of prevention [11].

Previous research has highlighted the importance of early sex education and its association with a decrease in risky sexual behaviors rather than with earlier onset and higher frequency of sexual relations. However, adolescents have reported that their schools do not provide a comfortable environment for sex education [11, 12]. These findings justify developing effective educational strategies to prevent risky sexual behaviors able to reach large numbers of adolescents.

The educational and health sectors and mass media experts have drawn up and implemented strategies to prevent risky sexual behavior in adolescents [13–17]. Numerous research studies found that formal sex education is more likely to influence safe behavior if narrowly focused, has an explicit behavioral message, and develops negotiation skills [14, 15]. Most of these studies were conducted in high schools and results showed that participants reduced their risky sexual behaviors [16, 17]. However, these interventions were subject to limited school physical spaces, tight schedules and the need for trained personnel.

In Mexico, the health and educational sectors have been implementing strategies aimed at increasing the knowledge of adolescents about sexual and reproductive health issues. The health sector is offering preventive programs consisting of educational workshops in primary care facilities. The rate of use is low because only 53 % of adolescents affiliated with public institutions use such services [18] which, in turn, affect its potential impact. Also, the educational sector provides lectures and materials on sexual and reproductive health in different school grades. Sex education starts in the sixth grade of elementary school and continues through high school. However, the quality of the contents and delivery of these lectures is unknown, and previous figures regarding the percentages of unwanted pregnancies, unsafe sex, and prevalence of sexually transmitted infections among Mexican adolescents signal the need to improve the effectiveness of current strategies.

The literature review within the Mexican context identified a small number of school-based interventions [11, 19, 20]. These studies have shown a moderate positive effect of increasing the knowledge related to HIV and emergency contraception [11, 19] and with the intention to use condoms and contraceptives [19]. However, there are several restrictions to implement these strategies on a large scale through traditional educational techniques including lack of trained personnel and physical spaces, whereas the time assigned to this activity might be short.

Recently, a new line of research promoting sexual health through digital technology has emerged. Adolescents are frequent users of the Internet, mobile phones and video games and it is beneficial to take advantage of these tools to implement educational strategies to prevent risky sexual behaviors. A recent systematic review included ten studies on this subject, seven of which took place in the U.S. [21]. Two of these studies significantly delayed the initiation of sexual intercourse. Seven interventions significantly influenced psychosocial outcomes such as self-efficacy for abstinence and condom use. Six studies increased the knowledge of participants about HIV, sexually transmitted infections and contraception. This type of intervention has some advantages: 1) Accessibility from any computer connected to the Internet. 2) Ability to reach large numbers of people (i.e., scalability). 3) Flexibility for participants to access the intervention content at their convenience. 4) Capability to automate most aspects of the study (e.g., evolutional surveys). 5) Privacy for answering sensitive questions. 6) Affordability because the provision of these services to additional individuals represents low marginal costs. 7) Plasticity because it allows use of diverse visual materials [22].

In Mexico, the number of Internet users is on the rise. Between 2011 and 2012 the growth rate was 8.8 %, reaching 40.9 million persons. Adolescents are the most frequent users [23]. Internet-based educational interventions have proven to be feasible, flexible, and efficient and able to reach a large number of persons [21, 22]. However, this type of intervention is rare in developing countries such as Mexico.

An educational strategy provided through an internet portal could reach large numbers of Mexican adolescents and be able to increase their knowledge of sexual and reproductive health topics. Although this kind of intervention should be included within the school-based activities, it has the advantage that it does not require significant organizational changes regarding school schedules, physical space, or highly trained personnel. Therefore, the objective of this paper is to present the design of an internet-based educational strategy to prevent risky sexual behaviors in Mexican adolescents.

## Methods

A field trial of one intervention and one comparison group with ex-ante and ex-post measurements will be conducted in two public secondary schools in the Iztapalapa Delegation in Mexico City. A delegation represents a geo-political area; Mexico City is divided into 16 delegations. Compared to the rest of the delegations, Iztapalapa has less favorable socioeconomic indicators [24]. The intervention will take place in one school and the second school will serve as a comparison group where we will observe the usual sex education being offered. The schools will be selected through simple random sampling from the list of public secondary schools in Iztapalapa. The study population will consist of adolescents between 14 and 15 years of age who are in the third year of secondary school and agree to participate through informed consent. Parents are also required to sign written informed consent.

The project was authorized by the National Research and Ethics Committee of the Mexican Institute of Social Security (R-2014-785-033), the Research and Ethics Committee of the Hospital Infantil de Mexico Federico Gómez (HIM 2015-066) and the General Directorate of Technical Secondary Education of the Secretary of Education.

### Preparation of the intervention

The conceptual framework for the intervention and educational materials was based on the model of the Information-Motivation-and-Behavioral Skills proposed by Fisher and Fisher [25]. This model emphasizes that adequate information regarding risky sexual behavior, changing the motivational component and training in behavioral skills can influence initiation and maintenance of protective sexual behaviors.

A research group comprised of a pediatrician, psychologist, and sociologist designed the educational materials. All research team members have experience in educational interventions. The academic basis for developing the materials were the books “You, Your Life, Your Dreams” developed by International Family Care [1] and “Your Future in Freedom, for Responsible Sexuality and Reproductive Health” published by the government of the Federal District and the Secretary of Education of the Federal District of Mexico City [26]. Investigators also took into account the statistics on sexually transmitted diseases and adolescent pregnancies from the World Health Organization, ENSANUT 2012, the National Registry of AIDS cases, and information from the Planned Parenthood Federation of America.

We designed teen-friendly educational materials. To accomplish this, we used “avatars” from www.bitstrips.com, which are comics for teenagers. We created two central characters (a teenage boy and a teenage girl) who present the information in dialogue form and talk about their own experiences as well as the experience of their friends.

Two experts in the area of adolescent sexual and reproductive health reviewed the educational materials. Twenty adolescents between 14 and 15 years of age participated in three rounds of pilot testing. Both the teenagers and their parents agreed to participate in this phase through verbal informed consent.

We used the snowball sampling technique to choose the teenagers who participated in the pilot phase, which included ten boys and ten girls, with an average grade point of 8.5 (range 8.0–9.0). Their families were representative of different structures (nuclear, single-parent, extended), and their parents had varying levels of education. Most adolescents (18) had not initiated sexual activity and only two reported being sexually active. Experts and teens were asked if the educational information was clear, understandable and if it used appropriate language for teens or if it was contradictory, uncomfortable, or uninteresting. The teens were asked to evaluate whether or not they liked each educational session (using a scale from 1─3 where 1 = No, I do not like it, 2 = I like it more or less, 3 = I like it) and to explain why they disliked the educational information. The final version of the educational material was produced taking into account the comments and suggestions of experts and teens who rated the information highly. Although most adolescents who participated in the pilot testing had not initiated their sexual life, they reported that the information was clear and interesting.

### Description and evaluation of the intervention

The study materials are available on the Internet web page (http://tu-ssexual.com), which can be accessed with the participant e-mail address and a centrally assigned password.

The intervention will include an introduction and four educational sessions (Table 1) and will last 1 month (one session per week) followed by a 3-month follow-up. Teens will be allowed to access a weekly session at any time and as often as he/she likes. However, the sessions will be consecutive so teens will not be able to access to the subsequent session without completing the previous one. Each session has an average duration of 1 h and ends with overview questions. Additionally, at the end of each session, teens will be asked what they like and dislike about the session. Answers will be recorded in the study database.

**Table 1:** Contents of the intervention

Session	Main topic	Contents
Introduction	Welcome	Welcome and general explanation of the organization of the website and instructions for its use.
		Invitation and compulsory automatic pass to answer the questionnaire that collects information on study variables.
1.	What I have to know before the first time or when it happened…?	1. What is dating? What is courtship? 2. Kisses, caresses and … what’s next? □ What is a sexual relationship? □ What is sexual pleasure? □ What is masturbation? □ What is sexual intercourse? □ Different sexual practices … 3. Having sex is the same for men as for women? 4. What should be a basis for a good relationship during courtship or dating? 5. How to recognize when your partner abuses you in order to not allow it (forms of abuse). 6. Self-esteem and its relation to self-care
2.	It is better to be informed and take care to prevent sexually transmitted infections	1. How sex can affect my health? Statistics of sexually transmitted infections (STIs) in adolescents in the world and in Mexico. 2. How can I realize when I or my partner has a sexually transmitted infection? 3. Are there medical tests to determine whether or not I have any type of sexually transmitted infection? 4. STIs affect women differently than men? 5. Are there vaccines to prevent STIs or treatments against them? 6. How I can protect myself from STIs? Ways to practice safe sex.
3.	It's better to know and care regarding HIV/AIDS and unwanted pregnancy.	1. HIV/AIDS: 1.1 How HIV/AIDS affects health. 1.2 Statistics on the prevalence of HIV/AIDS worldwide and in Mexico. 1.3. Ways of transmission of HIV/AIDS. 1.4 Common symptoms of people with HIV/AIDS: 1.5 Methods of detection and protection. 2. Is there any other risk in addition to STIs? And what if I get pregnant? What if my girlfriend gets pregnant? Teenage pregnancy statistics. Contraceptive methods: pros and cons. 3. Steps to use male and female condoms
4.	Preparing for a pleasant and safe sexual experience. Am I ready?	1. How can I negotiate condom use? Learning strategies for condom use negotiation 2. Tips for condom negotiation: □ at first intercourse □ at intercourse with a stable and casual partner □ when one of the two consumed alcohol □ when both are very excited 3. Make your own comics about how you would negotiate using a condom with your partner 4. E-sites on sexual and reproductive health

Furthermore, during the intervention and follow-up, booster e-mail messages will be sent to the teens of the intervention group. “Booster messages” will emphasize the main points of the educational sections and will be sent two to three times per week from the second week of the intervention.

Information on the study variables will be obtained through the Internet-based self-applied questionnaire and collected on three occasions: 1) when the adolescent enters into the study (baseline), 2) once the intervention is completed (at 1 month) and 3) after 3 months of follow-up (fourth month).

In the control group, teens will continue the usual school-provided sex education and will be invited to answer the Internet-based self-applied questionnaire at the same time as the intervention group. Furthermore, at the conclusion of the study, teens from the control group will also receive access to the educational sessions.

### Recruitment and retention strategies

To improve recruitment and retention of teens in the study, we will implement strategies focused on teens, their teachers and parents. Recruitment strategies will incorporate provision of information and reinforcement of the potential benefits of the intervention. Retention strategies will include the following: 1) e-mail reminders of each session; 2) placing reminders with brief thematic description of the weekly session in classrooms; 3) follow-up e-mail messages about technical and other problems regarding each learning session and solutions to such problems; 4) parent and teacher involvement in motivating adolescents’ participation.

### Study variables

There will be three outcome variables:

1. Knowledge of sexually transmitted infections as measured by the scale validated in Mexican teens by Robles-Montijo and Diaz-Loving [27]. This scale consists of 23 items with a response format “true”, “false” and I don’t know”. The sum of correct answers ranges from 0 (absence of knowledge) to 23 (high knowledge).
2. Attitudes regarding condom use will be measured by the UCLA Multidimensional Condom Attitudes Scale [28]. This scale consists of 25 items and assesses five independent factors: (i) reliability and effectiveness of condoms, (ii) pleasure associated with condom use, (iii) stigma attached to being a condom user, (iv) embarrassment associated with the negotiation and use of a condom, and (v) embarrassment associated with the purchase of a condom. The scale was previously validated in Mexico [29] and has a Likert-type response format with values ranging from 1 (strongly disagree) to 7 (strongly agree) and with an intermediate value of 4 (undecided). Some questions are worded negatively indicating a negative attitude, so the score has to be reversed. Each domain is scored separately producing a score per domain. The domain rating is calculated as the sum of the items in the domain.
3. Self-efficacy toward consistent condom use will be assessed by the seven measures proposed by Fishbein and colleagues [30] and then translated into Mexican Spanish by Robles-Montijo & Diaz-Loving [27]. These include one direct and six indirect measures toward consistent condom use. The direct measure will be obtained by the response to the item ‘Many different things can get in the way of using a condom when people have sex. How sure are you that you can use a condom every time you have vaginal sex with your main partner?’ The response is coded on a 1–7 Likert scale with ‘very sure I cannot’ and ‘very sure I can’ as endpoints. The indirect measure of self-efficacy toward consistent condom use will be obtained using six items. The first five had the commonality of ‘how sure are you that you can (could) always use a condom for vaginal sex?’ The conditions were: (1) when he/she has been drinking or using drugs, (2) when you have been drinking or using drugs, (3) when he/she is very sexually excited, (4) when you are very sexually excited, and (5) when he/she does not feel like using a condom. The final item is focused on an abstinence self-efficacy: ‘what if you and your main partner want to have sex and a condom is not available? How sure are you that you can put off having vaginal sex with your main partner until you or he/she can get one?’ All responses to indirect measures are on a 0─10 scale format where 0 indicated ‘absolutely sure you cannot’ and 10 ‘absolutely sure you can’ [30].

We will also measure and statistically control for covariates related to teens’ preventive behaviors. These covariates include parental characteristics such as parental education, occupation, family structure and parental communication about risky sexual behaviors; information on risky sexual behaviors obtained through the mass media in the 3 months prior to each study evaluation, and the teens’ general characteristics such as sex, age, school performance (average grade point for the past year), substance use (tobacco, alcohol and drugs) and social desirability.

Parental communication about risky sexual behaviors will be measured by the scale designed and validated in Mexican adolescents by Robles-Montijo & Diaz-Loving [27]. This scale has ten items, which reflect communication about sex, sexually transmitted infections, alcohol and drug consumption, unwanted pregnancy, use of birth control, abortion, and male and female condoms. Responses are coded on a 1─3 Likert format scale: 1 (never), 2 (occasionally) and 3 (always). The total sum score varies from 10 (no communication) to 30 (strong communication).

Social desirability will be measured with the “Lie scale” of the Eysenck Personality Questionnaire. This questionnaire measures three domains of personality (psychoticism, neuroticism and extroversion) and includes a social desirability or ‘Lie scale’ that measures attempts to answer in a socially desirable manner. The Lie scale consists of 20 items with dichotomous response format (Yes or No). The responses sum score ranges from 0 (low social desirability) to 20 (high social desirability). This scale was validated in Mexican children between 12 and 15 years of age [31].

Furthermore, we will also measure and compare (between intervention and control group) the variables related to the teen’s sexual behaviors. These behaviors include history of first intercourse, number of sexual partners, age of current or most recent sexual partner, time elapsed after the last intercourse, and condom use during the first and last intercourse. We did not consider these variables as outcomes because previous studies reported that only ~20 % of Mexican adolescents from the third year of secondary school are sexually active [32, 33] and because the primary objective of this study is to design an intervention to prevent risky sexual behaviors.

### Sample size

Sample size was calculated using the software “A-priori Sample Size Calculator for Multiple Regression” (available at: http://www.danielsoper.com/statcalc3/calc.aspx?id=1). Effect of the intervention will be accessed by multiple regression analysis considering possible confounders due to the quasi-experimental nature of the study design. We considered the medium size effect for F-test (f2) of 0.15, alpha of 0.05, beta of 0.90, and 15 covariates. The number of teens by group, assuming a drop-out rate of 25 %, will be 200.

### Statistical analysis

The unit of analysis and inference will be an adolescent. Comparison of the characteristics of teens and their parents between intervention and control groups will be made using the chi-square test for categorical variables and Student t-test for continuous variables to describe the study population and ascertain similarity of both groups at baseline measure.

Scores for each outcome variable will be calculated according to the scoring algorithms. Differences in baseline and post-intervention stages for outcome variables will be compared within each group. Differences between groups will be compared using the differences-in-differences (D-in-D) estimator [34]. The generalized linear model (GLM) will be used to assess changes in each outcome variable controlling for baseline measures and study covariates. GLM methods allow for modeling the correlation of the repeated observations on the same subjects [35].

The analysis will be performed according to the intention-to-treat approach. Missing measurements due to a participant’s withdrawal from the study will be replaced with the most recent observations; p value <0.05 will be considered as statistically significant. The analysis will be performed using Stata v.11.0 statistical software (Stata 11.0, Stata Corp; College Station, TX).

## Discussion

Mexico has a high proportion of adolescents; almost one in five persons is between 10 and 19 years of age, accounting for 22.4 million persons [36]. Additionally, the rate of adolescent unintended pregnancy and sexually transmitted diseases is high. The National Survey of Demographic Dynamics [37] reported that in 2014 there were 77 births/1000 girls aged 15─19 years, whereas in 2009 there were 70.9 births. Mexico is at the top of the list for teenage pregnancy among member countries of the Organization for Economic Cooperation and Development. Adolescents also have infrequent contact with preventive health services [18] and receive inadequate sex and reproductive health education at school. In this context, it is justifiable to support the efforts of the health and education sectors in Mexico by testing innovative interventions to improve the sexual and reproductive health of adolescents.

The proposed intervention has several strengths. (i) Educational materials for this intervention are visually attractive and culturally adopted. (ii) The Internet will be the vehicle to deliver the intervention because it is popular and widely accepted by adolescents and has multiple advantages that we addressed in the Introduction. (iii) The intervention comprises recruitment and retention strategies as previous educational Internet-based interventions have reported rates of participant attrition >25 % [38]. Therefore, use of retention strategies is crucial as the retention of participants is a parameter of the internal validity, feasibility, and acceptability of the study. Furthermore, we anticipated the dropout rate in sample size estimation. (iv) The intervention will also include booster strategies as previous studies found major effects in the intervention groups that incorporated such strategies compared with those that did not [39]. (v) Finally, results of the intervention will be statistically controlled for potential confounding variables including “social desirability”. Previous studies in the field of health-related behaviors have identified the presence of social desirability bias in self-reports of respondents. Social desirability refers to the systematic tendency of individuals to provide a better image of themselves, reporting responses perceived as more socially acceptable [40]. It has been identified that social desirability bias in the participants’ response is similar in the printed paper surveys and anonymous online surveys [41]. Therefore, measurement and control of this bias is recommended [40]. Several scales to assess social desirability exist; however, most were designed and validated for adults. According to our knowledge, only one scale has been designed and validated in English for measuring social desirability in children and adolescents, which is the Children’s Social Desirability Scale [42]. However, this scale is extensive (48 items) and has not been validated in Spanish. At the same time, the “Lie scale” of the “Junior Eysenck Personality Questionnaire” was validated in 25 countries including Mexico. Application of this scale showed that Mexican children and adolescents express greater social desirability compared to U.S. children [31]. Taking into account this background, we decided to measure the desirability bias using the “Lie scale” as well as to control this bias in the statistical analysis.

Also, several limitations can be noted. (i) The proposed study design is ‘quasi-experimental’ with one intervention and one comparison group, with ex-ante and ex-post measurements. Therefore, the assignment of teens to the intervention and control groups will not be random. This could lead to interpretation problems if baseline measurements differ between groups. Furthermore, in order to address baseline differences between intervention and control group we proposed to perform the D-in-D estimator in the framework of a generalized linear regression model. (2) Due to financial feasibility issues we could not use an extensive (6 months or longer) follow-up and evaluate health-related outcomes such as pregnancies and sexually transmitted infections.

In conclusion, it is justifiable to design and evaluate an Internet-based educational strategy to prevent risky sexual behaviors in Mexican adolescents in order to provide a new, large scale, easily implemented and preventive tool.
